# Bilateral Renal Lymphangiomatosis: A Case Report and Literature Review

**DOI:** 10.7759/cureus.58180

**Published:** 2024-04-13

**Authors:** Ahmad M Abualrub, Waleed M Malhes, Mohammad H Shehadeh, Fadi H Omari, Anas R Tuqan, Shaima Ishtawi, Tareq Hindi

**Affiliations:** 1 Department of Medicine, Al-Quds University, Jerusalem, PSE; 2 Department of Pediatrics, Palestine Medical Complex, Ramallah, PSE; 3 Department of Pediatric Nephrology, Augusta Victoria Hospital, Jerusalem, PSE

**Keywords:** case report, hypertension, computed tomography, children, renal lymphangiomatosis

## Abstract

Renal lymphangiomatosis is a rare congenital condition characterized by the abnormal development of lymphatic channels in the kidney, resulting in cystic dilatations. While more commonly observed in children, it can occur in adults but is extremely rare. Clinical manifestations range from asymptomatic cases to symptoms such as abdominal pain, hypertension, and renal dysfunction. In this case report, we present a rare case of bilateral renal lymphangiomatosis in an eight-year-old male with high blood pressure. Renal ultrasound revealed bilateral kidney enlargement and perinephric hypoechoic collections with septations consistent with lymphangiomatosis. The diagnosis was confirmed through CT imaging which shows bilateral non-enhancing perinephric collections. As a part of the patient's management plan, bilateral perinephric cystic lesions were successfully aspirated without complications. In conclusion, accurate diagnosis is crucial for appropriate management decisions, and treatment primarily focuses on conservative measures to manage associated hypertension, reduce lymphatic fluid accumulation, and alleviate pain, reserving invasive interventions for severe cases or complications.

## Introduction

Lymphangiectasia, also referred to as lymphangioma or lymphangiomatosis, is an uncommon condition that affects the mesenchymal tissues. It is characterized by abnormal development of lymphatic channels ​[1]. The lymphatic tissue surrounding the kidneys does not form proper connections with the rest of the lymphatic system. As a result, the lymphatic channels around the kidneys become enlarged, resulting in the formation of a cystic mass that can be either unilocular or multilocular ​[2]. While lymphangioma is typically observed in children, it rarely occurs in adults ​[3]. The most prevalent locations for lymphangioma are the neck (75%) and axillary region (20%), although it can also manifest in other locations, such as the retroperitoneum, mediastinum, mesentery, omentum, colon, and pelvis ​[4]. Lymphangioma involving the kidney is an exceptionally uncommon occurrence. To our knowledge, only 104 cases of renal lymphangiectasia have been reported worldwide ​[5].

In this report, we present a rare case of bilateral renal lymphangiomatosis and provide insights into its diagnosis and management with a literature review.

## Case presentation

An eight-year-old male presented to the emergency room due to a high blood pressure reading at home. This patient was doing well until four days later when the patient started complaining of headaches. Before coming to the hospital, the parents recorded the patient's blood pressure, which was 164/102 mmHg. The patient did not report symptoms such as flank pain, hematuria, dysuria, constipation, or diarrhea. Upon examination, his blood pressure was 165/98 mmHg, so the patient was admitted to the pediatric floor for further evaluation. He has a documented history of developmental delay, spastic quadriplegia, and convulsion disorder, suspected to be associated with congenital cytomegalovirus infection. Whole exome sequencing (WES) to identify mutations was done and showed no mutations. Drug history includes Baclofen 10 mg bid and Valproic acid 200 mg bid. Past surgical, perinatal, and family history are unremarkable. There is no consanguinity.

Physical examination revealed normal findings, except for hypertonicity in the lower limbs since birth. Subsequent blood pressure readings remained elevated, with the highest recorded value being 172/101 mmHg. Laboratory investigations including complete blood count, kidney function tests, and liver function tests were within normal limits (Table 1). Urinalysis was done and revealed no abnormalities (Table 2).

**Table 1:** Laboratory tests of the patient

Test	Result	Reference range
Hemoglobin (HGB)	11.6 g/dL	11.5-15.5 g/dL
Hematocrit (HCT)	40.7%	34-40%
Mean cell volume (MCV)	74.7 fL	76-80 fL
Mean cell hemoglobin concentration (MCHC)	31.2 g/dL	31-35 g/dL
White blood cells (WBC)	11.9 K/uL	4.5-13.5 K/uL
Platelets count	371 K/uL	150-450 K/uL
Aspartate aminotransferase (AST)	55 U/L	0-50 U/L
Aspartate transaminase (ALT)	34 U/L	0-41 U/L
Creatinine	0.7 mg/dL	0.7-1.2 mg/dL
Blood urea nitrogen (BUN)	10.4 mg/dL	6-20 mg/dL
BUN/creatinine ratio	14	9-20

**Table 2:** Urinalysis of the patient

Test	Result	Reference range
Specific gravity	1.015	1.003-1.029
pH	6	4.5-7.8
Urine color	Yellow	Yellow
Protein	Trace	Negative
Glucose	Negative	Negative
Ketones	Negative	Negative
Occult blood	Negative	Negative
Bilirubin	Negative	Negative
Nitrite	Negative	Negative

**Figure 1:**
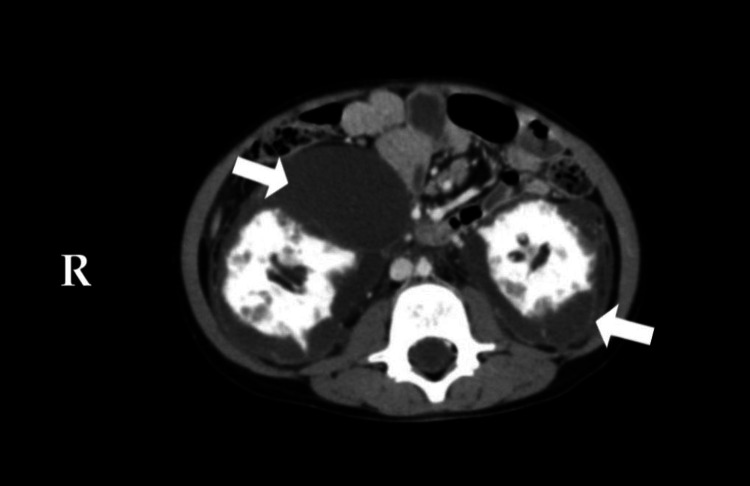
Contrast-enhanced CT of the abdomen (axial views): there are bilateral non-enhancing perinephric collections (white arrows). Both kidneys have normal perfusion

**Figure 2:**
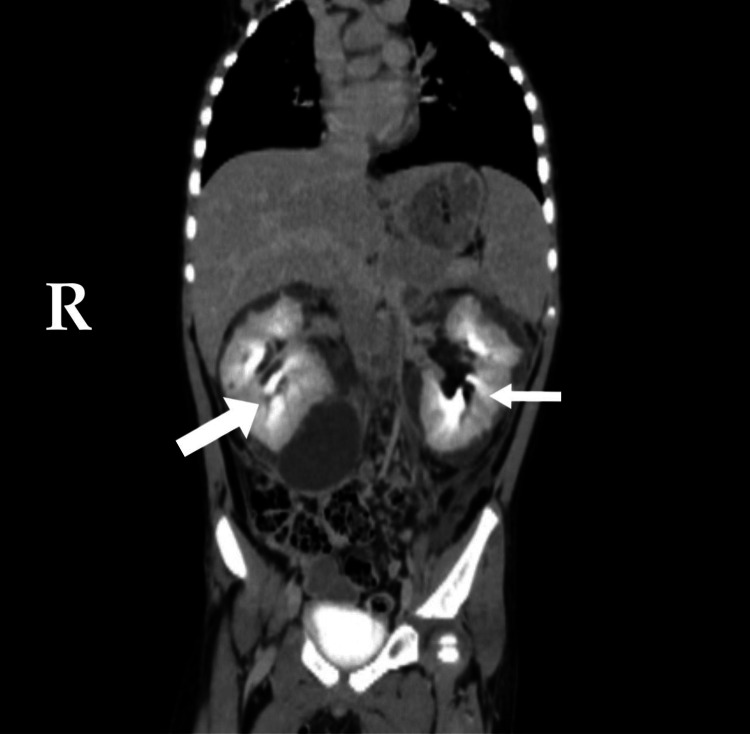
Contrast-enhanced CT of the abdomen (coronal view). There is compression of the renal parenchyma bilaterally (white arrows)

**Figure 3:**
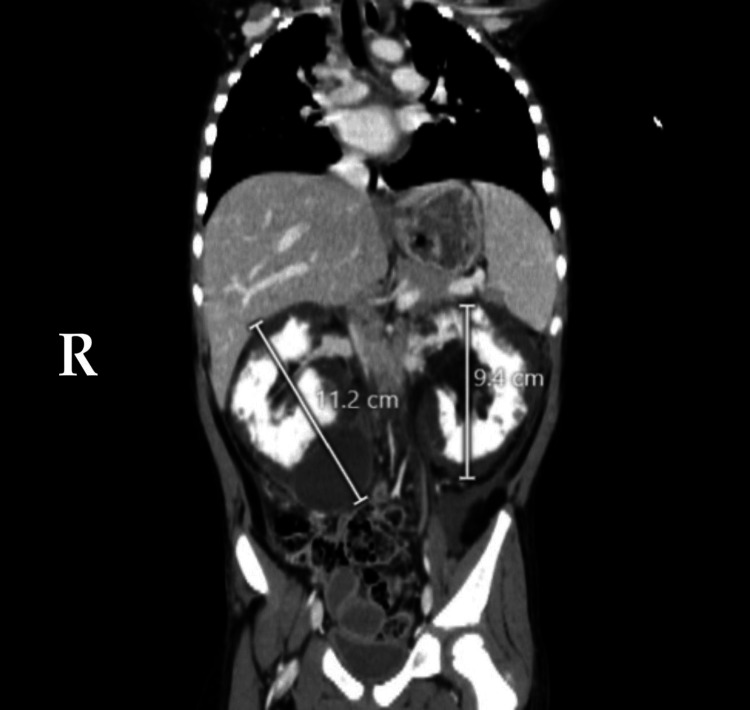
Contrast-enhanced CT of the abdomen (coronal view). Large fluid density collections are noted in bilateral perirenal regions, measuring 11.2 cm on the right side and 9.4 cm on the left side in the maximum craniocaudal (CC) dimension

A renal ultrasound examination revealed bilateral enlargement of the kidneys, with the right kidney measuring 10.3 cm and the left kidney measuring 11 cm. Additionally, there are bilateral perinephric hypoechoic collections with septations raising the question of possible lymphangiomatosis, abscess, or hemorrhage. Subsequent abdominal computed tomography (CT) scans, both with and without contrast, confirmed the presence of bilateral non-enhancing perinephric collections with average density from 0 Hounsfield units (HU) to 10 HU (Figure 1), along with compression of the renal parenchyma bilaterally (Figures 2, 3). However, contrast excretion into the collecting system was normal, and no abnormalities were detected in other abdominal structures. These findings on CT were consistent with a diagnosis of bilateral renal lymphangiomatosis.

Following the confirmation of renal lymphangiomatosis, the patient was referred to a pediatric urologist for further management. Aspiration of the bilateral perinephric cystic lesions was performed without complications. Follow-up after two weeks revealed a resolution of high blood pressure, patient has no other complaints.

## Discussion

Renal lymphangiomatosis is a rare benign pathology characterized by the accumulation of lymphatic fluid in the perirenal, parapelvic, and intrarenal lymphatics, leading to the formation of unilocular or multilocular cystic dilatations ​[6]. It is a congenital disease that can be unilateral or bilateral and is caused by obstruction of the lymphatic system during the developmental period ​[3]​. However, the pathophysiology of renal lymphangiomatosis remains poorly understood. It is hypothesized that there is a lack of communication between the renal lymphatic vessels and the larger retroperitoneal lymphatics ​[7]. Renal lymphangiomatosis has been observed in both children and adults, affecting individuals across various age groups with no gender predilection [8].

Mani et al. documented a case involving an eight-year-old male patient with renal lymphangiomatosis who presented with gradually progressive bilateral flank swelling but who was otherwise asymptomatic [2]. The clinical presentation of renal lymphangiomatosis varies, ranging from asymptomatic cases incidentally discovered to those presenting with abdominal pain, abdominal mass, hematuria, flank or back pain, proteinuria, renin-dependent hypertension, fatigue, and weight loss​ [9,10]. Abdominal pain, abdominal distension (ascites), and hypertension are the most commonly observed symptoms in pediatric patients ​[3]. Ascites were found to be the only statistically significant difference in presentation between pediatric and adult age groups ​[5]. Complications that can be encountered include cyst-related complications such as rupture, hemorrhage, and infection​ [6]. Additionally, complications related to mass effects can occur, such as renal vein thrombosis, obstructive uropathy, and deterioration of renal function ​[3,10]. It is important to note that pregnancy can worsen the disease by increasing the fluid content within cysts ​[11].

Diagnosing renal lymphangiomatosis involves a combination of clinical evaluation and imaging studies, such as ultrasound, CT, and magnetic resonance imaging (MRI), which demonstrate classical features that enable a confident diagnosis of renal lymphangiomatosis ​[10]. In some cases, needle aspiration of chylous fluid from perinephric collections may be necessary for confirmation ​[12]. Ultrasound is often used as a primary diagnostic tool due to its ease of use and lack of side effects ​[10]. In the majority of cases, contrast-enhanced computerized tomography (CECT) is diagnostic ​[13]. On CECT, lymphatic dilations appear as hypodense structures (0-10 HU), non-enhancing, non-communicating structures surrounding the kidneys and renal pelvis ​[13]. MRI can provide additional information regarding tumor size and extension ​[10]. MRI reveals a hypointense appearance in T1-weighted images and a hyperintense appearance in T2-weighted images, with a reversal of the cortico-medullary intensity ​[13]. In ambiguous cases, histopathological correlation through needle biopsy may be required to confirm the diagnosis ​[5].

Alshanafey et al. reported a case involving a 12-year-old boy who presented with progressive abdominal distension and flank pain persisting for three months. The initial provisional diagnosis prior to his referral was polycystic kidney disease [5]. The two most frequently encountered differential diagnoses with a high misdiagnosis rate are polycystic kidneys and hydronephrosis ​[10]. Hydronephrosis is characterized by enlargement of the collecting system, with or without a dilated ureter ​[14]. Autosomal dominant polycystic kidney disease (ADPKD), unlike renal lymphangiomatosis, typically shows a widespread abnormality of renal parenchyma ​[15]. Moreover, various tumors, including liposarcoma, leiomyosarcoma, fibrosarcoma, malignant teratomas, and multilocular cystic nephroma, may mimic the cystic or necrotic appearance of renal lymphangiomatosis ​[15]. However, these tumors often have significant solid components, distinguishing them from the purely cystic nature of lymphangiomatosis ​[15], and conditions such as abscesses and urinomas might also resemble renal lymphangiomatosis on imaging. However, it is generally possible to differentiate these conditions from renal lymphangioma based on the patient's clinical history, normal laboratory results, and specific imaging characteristics that indicate the involvement of the perirenal and parapelvic areas without affecting the renal parenchyma ​[15]. Angiomyolipoma is another differential diagnosis ​[10]​ that, although it may present cystic features similar to lymphangiomatosis, is usually identifiable by the presence of fat within the lesions and enhanced solid components in imaging studies ​[16].

Treatment for renal lymphangiomatosis is typically conservative due to the benign nature of the condition. The mainstay of therapy includes antihypertensive medication to manage associated hypertension, diuretics to help reduce lymphatic fluid accumulation, and painkillers to alleviate associated pain​ [17,18].​ In cases where conservative management is insufficient, especially when complications such as infection or bleeding occur, more invasive interventions may be considered ​[5]. These include percutaneous drainage to remove excess lymphatic fluid, which has a high failure rate due to the presence of multiple loculations ​[19]. Sclerotherapy obliterates abnormal lymphatic vessels but requires multiple sessions ​[13]. Marsupialization creates an external drainage pathway for the lymphatic fluid ​[20]​ and nephrectomy, which is considered in severe cases where the kidney function is compromised or if the condition is unilateral and causes significant symptoms ​[5]. It is important to note that no definitive treatment protocol exists because of the rarity of this condition. Each case may require a tailored approach based on the severity of the symptoms and the presence of complications. The approach taken in this case, involving aspiration of the cystic lesions, appears to be a safe and effective initial intervention. However, long-term follow-up is essential to monitor for potential recurrence or complications and to assess the need for additional interventions such as sclerotherapy or surgical resection in the event of symptomatic or progressive disease.

The rarity of renal lymphangiomatosis poses a challenge for clinicians. This scarcity highlights the importance of case reports in contributing to a collective understanding of the disease. Each case provides valuable insights into the clinical presentation, imaging findings, complications, and differential diagnosis of this rare entity, thereby enriching the knowledge base and aiding the development of management guidelines.

## Conclusions

In conclusion, our case report highlights the rarity of bilateral renal lymphangiomatosis and the challenges in its diagnosis and management. Through clinical evaluation and imaging studies, we were able to confidently diagnose the condition. Management focuses on symptom control and monitoring for complications. Conservative treatment with anti-hypertensive medication and diuretics was considered, while invasive interventions were reserved for complications. This case emphasizes the need for increased awareness and understanding of renal lymphangiomatosis to facilitate early detection and appropriate therapeutic strategies to prevent complications.
